# Synthesis of Polystyrene Particles with Precisely Controlled Degree of Concaveness

**DOI:** 10.3390/polym10040458

**Published:** 2018-04-21

**Authors:** Wenhua Jing, Sinan Du, Zexin Zhang

**Affiliations:** 1 Centre for Soft Condensed Matter Physics and Interdisciplinary Research, Soochow University, Suzhou 215006, China; 18896782986@163.com (W.J.); polymerdsn@163.com (S.D.); 2 State and Local Joint Engineering Laboratory for Novel Functional Polymeric Materials, College of Chemistry, Chemical Engineering and Materials Science, Soochow University, Suzhou 215123, China

**Keywords:** concave particle, seeded emulsion polymerization, good solvent, buckling, self-assembly, depletion interaction

## Abstract

Shape is an essential property of polymeric particles. Herein, we propose a simple method to synthesize polymeric particles with a well-controlled concave shape. Our method takes advantage of the powerful seeded emulsion polymerization strategy with the well-known principle of “like dissolves like” in solvent chemistry. We first prepared polystyrene (PS) particles with a single dimple by seeded emulsion polymerization. Then the dimpled PS particles were dispersed in a dimethylformamide (DMF) and water mixture. Consequently, the non-crosslinked polymer chains inside the particle were dissolved by DMF, a good solvent for PS, and the PS chains migrated out of the particle, causing buckling of the dimple and enlargement of the concave. By systematic change of the fraction of DMF in the solvent mixture, we changed the amount of the dissolved PS chains, and achieved polymeric particles with precisely tuned degree of concaveness. These concave particles were found to readily self-assemble, driven by polymer-induced depletion interaction. The concave PS particles reported here provide potential building blocks for self-assembled polymeric materials, and new model systems for condensed matter research.

## 1. Introduction

Anisotropic polymeric particles have attracted great attention due to their importance in both fundamental research and potential applications. On the one hand, anisotropic particles can be used as experimental models to study the structure and dynamics of condensed states such as crystal and liquid crystal. On the other hand, anisotropic particles have begun to be used as novel materials in molecular recognition, self-assembly, and Pickering emulsion, to name a few applications [1,2,3]. With the development of synthesis strategies, a variety of polymeric particles with anisotropic shapes have been synthesized [4,5,6,7,8], but precise control of the shape is still very challenging. Shape is one of the most basic and important properties of polymeric particles. Common anisotropic shapes include concave, mushroom, dumbbell, snowman, ellipsoid, etc. [9,10,11,12]. Among them, concave-shaped particles have shown great potential in orientational self-assembly, targeted bonding and nano-/microchemical reactors, and hence attracted intensive research effort in recent years [13,14,15,16]. In general, the preparation of concave polymeric particles falls in the following three categories: microfluidics, solvent swelling and core-shell shrinkage [15,16,17,18,19]. These methods have disadvantages, such as sophisticated procedures, low-throughput production, and polydispersity in size and/or shape. Recently, seeded emulsion polymerization has made a breakthrough in the preparation of anisotropic particles, enabling the preparation of monodispersed concave particles in large quantity [20,21,22]. However, a simple method for systematic control of the degree of concaveness is still rare, which not only greatly limits the applications of the particle as polymeric materials, but also prevents the particle from being used as a model system where a tunable degree of concaveness is crucial for any quantitative research [22,23].

In this paper, a simple method for the synthesis of polymeric particles with a controlled degree of concaveness is proposed. PS particle with one single dimple is synthesized by a well-documented seeded emulsion polymerization procedure reported by our group and others [23,24]. Then the particles are dispersed in a solvent mixture of DMF/Water, a good—poor solvent combination for PS. Some of the non-crosslinked PS chains inside the particles are dissolved by DMF and removed by subsequent wash, leading to buckling of the dimpled particles [25]. By simply change the fraction of DMF in the solvent mixture, we can control the amount of PS dissolved. And consequently, we can precisely tune the buckling process to produce polymeric concave particles with well-defined degree of concaveness. Moreover, the polymeric concave particles are found to self-assemble, driven by depletion attraction when their size and shape match.

## 2. Materials and Methods 

### 2.1. Materials

Styrene (St, GR, *M*_w_ = 104.15 g/mol), divinylbenzene (DVB, 55%, *M*_w_ = 130.19 g/mol), azobisisobutyronitrile (AIBN, AR, *M*_w_ = 164.21 g/mol, recrystallized before use), dimethylformamide (DMF, GC, >99%, *M*_w_ = 73.09 g/mol) were purchased from Aladdin (Shanghai, China). Polyethylene alcohol (PVA, AR, *M*_w_ = 41,240 g/mol, alcoholysis degree of 88%) were purchased from Alfa Aesar (Haverhill, MA, USA), and polyvinylpyrrolidone (PVP, *M*_w_ = 40,000 g/mol), poly(ethylene oxide) (PEO, *M*_w_ = 600,000 g/mol) were purchased from Sigma (St. Louis, MO, USA).

### 2.2. Synthesis of PS Dimple Particles with Controllable Concave

#### 2.2.1. Synthesis of PS Dimple Particles

Seven and a half grams of styrene monomer were dispersed in 22.5 mL of ethanol, and then 135 μL of cross-linker, DVB was added. The mixture was stirred well under the protection of nitrogen gas, and was labeled as solution 1. In a separate flask, 7.5 g of styrene monomer was dispersed in 22.5 mL of ethanol, followed by adding 0.16 g of AIBN and 1.6 g of PVP, and the resulting mixture was labeled as solution 2. Here the PVP was a stabilizer that can increase the relative viscosity of the solution and prevent the sedimentation of PS particles. Solution 2 was stirred under a nitrogen atmosphere for 30 min, followed by heating to 73 °C. After the solution became turbid, solution 1 was slowly added into solution 2 at a rate of 0.25 mL/min by a peristaltic pump. After the addition was completed, the reaction continued for 24 h. The as-synthesized particles were repeatedly washed by centrifugation–decantation in deionized water and then dried in a vacuum oven at 40 °C for 24 h.

#### 2.2.2. Systematic Control of the Degree of Concaveness

Twenty milligrams of the PS dimple particle was dispersed in a mixture of 30 mL DMF and deionized water with different volume ratio (*R* = 0:10, 2:8, 4:6, 6:4, 8:2, 9:1, with *R* = *V*_DMF_/*V*_water_). To dissolve the non-crosslinked PS chains, the particle dispersion was slowly (5 rpm) rotated on a rolling incubator (Qilin-Bell, Haimen, Jiangsu, China) at room temperature for desired time. The slow nature of polymer dissolution and the cross-linked surface layer of the dimple PS particles made the dissolution and transportation of the polymer chains difficult. By systematic change of the dissolution time we found that 21 days of dissolution always yields reproducible results, regardless of the mixing ratios of the DMF and water (see Supplementary Materials for more discussions on the dissolution of the polymer). So we applied the 21-day rule to all the samples. The resultant particles were washed by centrifugation–decantation in deionized water three times.

#### 2.2.3. Self-Assembly of Concave Particles

2 mg of PS concave particles (diameter: 3.0 μm) and 8 mg of PS spheres (diameter: 1.5 μm or 3.0 μm) were dispersed in 1 mL of PEO solution (PEO concentration, 0.5 g/L). The solvent for the solution was deionized water and no salt was added. The addition of PEO induced depletion interaction between the particles to turn on the assembly of the particles.

### 2.3. Characterization

#### 2.3.1. Optical Microscope

Mixture of the concave particle suspension and the PEO solution was sealed with UV glue between two cover glasses (Fisher, Hampton, NH, USA). The assembly behavior of the particles in the sample was observed and recorded by an inverted optical microscope (Observer A1, Zeiss, Oberkochen, Germany).

#### 2.3.2. Scanning Electron Microscope (SEM)

The concave particles were examined with a scanning electron microscope (SEM, SU8010, Hitachi, Tokyo, Japan) after the sample was sputter-coated with gold. The size information of the particle was obtained by image analysis using open-source software, ImageJ (NIH, Bethesda, MD, USA).

#### 2.3.3. Infrared Spectroscopy (IR) and 1H Nuclear Magnetic Resonance (NMR)

The dissolved material from the PS dimple particle was characterized with IR and NMR. The IR spectra were recorded on a VERTEX 70 (Bruker, Karlsruhe, Germany) infrared spectrometer, and the NMR spectra were recorded on an INOVA 400 MHz (Varian, Palo Alto, CA, USA) spectrometer using deuterated chloroform (CDCl_3_) as solvent. 

## 3. Results and Discussions

### 3.1. Formation of PS Particles with Controlled Degree of Concaveness

**Figure 1:**
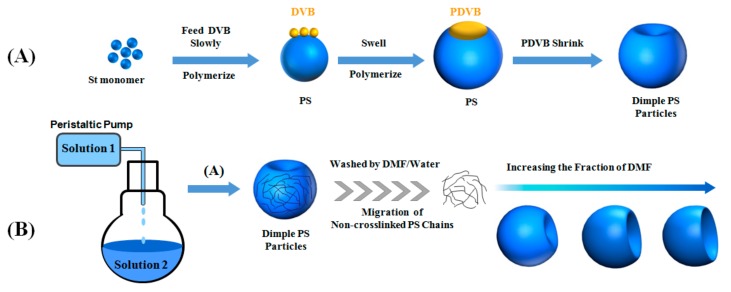
Schematic of (**A**) Polymerization of styrene with programmed feeding of cross-liker, DVB to form dimple PS particles; (**B**) formation of PS particles with controlled degree of concaveness. Solution 1: styrene, DVB and ethanol; Solution 2: styrene, AIBN, PVP and ethanol.

A schematic representation of the synthesis is summarized in Figure 1. The method combines the powerful seeded emulsion polymerization with the well-known principle of “like dissolves like,” i.e., polymers tend to dissolve in solvents of similar polarity. The polystyrene (PS) particles with a single dimple are prepared by seeded emulsion polymerization [26,27]. Then the PS dimple particles are dispersed in a solvent mixture of DMF/Water. Here the choice of solvents is based on three facts. Firstly, DMF is miscible with water and can form a one-phase solvent for the PS particle. Secondly, DMF has a boiling point of ~152 °C and is less volatile than other common organic solvents, making it perfect for our long-term dissolution experiments. Thirdly and more importantly, the solubility parameter of PS is very close to that of DMF, but is very different from that of water (with the value of PS, 16.6–20.2 (Mpa)^0.5^, DMF, 24.7 (Mpa)^0.5^, and water, 48.1 (Mpa)^0.5^) [28,29]. According to the principle of “like dissolves like” principle, PS can be dissolved by DMF, but to a much less degree by water. Hence, by systematically changing the mixing ratio of DMF and water, we control the amount of PS dissolved. After the dissolved PS chains were removed, the particles were found to buckle due to material loss. And as a result, the degrees of concaveness are precisely tuned by this dissolution method [24].

**Figure 2:**
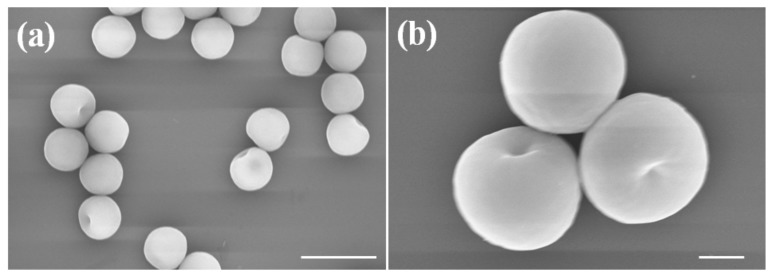
SEM images of PS dimple particles. Scale bar: (**a**) 5 μm; (**b**) 2 μm.

The dimple particles are synthesized by seeded emulsion polymerization with programmed feeding of the cross-linker. From the SEM images, uniform-sized particles with one single dimple are observed (Figure 2). The diameter of the dimpled particle and the size of the concave (maximum length of the open end) are found by image analysis to be 3.0 ± 0.1 μm and 500 ± 20 nm, respectively. The results are consistent with our previous work on the synthesis of anisotropic particles [23,25].

### 3.2. Synthesis and Characterization of PS Particles with Controllable Concave

**Figure 3:**
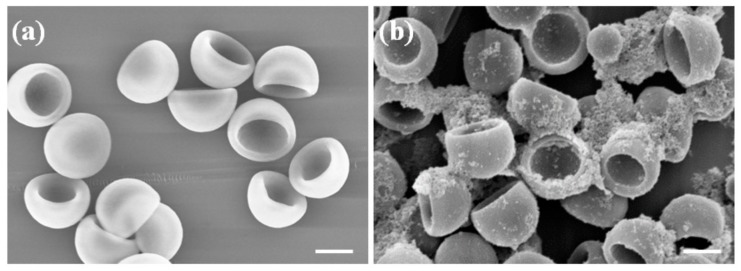
SEM images of the concave PS particles with (**a**) and without (**b**) the centrifugation and wash. The fluffy materials sticking to the dimple particles in (**b**) are believed to be the dissolved PS chains. Scale bar: 2 μm.

**Figure 4:**
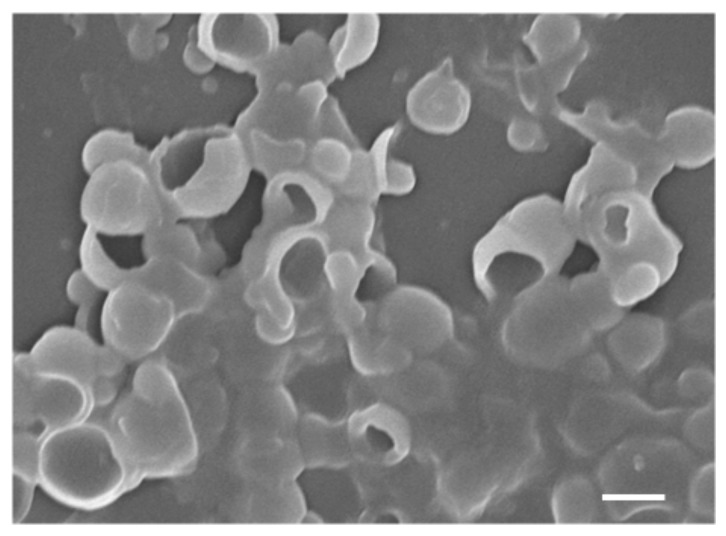
SEM image of the concave PS particles after exposure to pure DMF. Most of the particles are dissolved and collapsed by the good solvent DMF. Scale bar: 2 μm.

Shape of polymeric particle is fixed once they are synthesized, mainly due to the fact that common polymers, such as PS and PDVB, have higher glass transition temperatures than room temperature. The key procedure in our synthesis is to induce the shape change in a controlled manner. This is achieved by a clever choice of solvent mixtures. We choose a good solvent for PS, DMF, to soften the particle, and more importantly, to dissolve the non-crosslinked PS chains in the particle [18,29]. After removing the dissolved PS chains by centrifugation and wash, we found the particles buckled and the concaves enhanced due to the loss of the material. To demonstrate this dissolution effect, we examine the dimple PS particles with and without the centrifugation and wash. The results are shown in the SEM images in Figure 3. The fluffy solid material, distributed randomly on or near the unwashed particles, is believed to be the dissolved PS chains (Figure 3b). These polymer chains were collected during the wash and centrifugation step. By drying supernatant in the centrifugation tube after the particles are separated, we obtain the same fluffy solid material. We then checked the material by infrared spectroscopy and ^1^H NMR, and it is indeed polystyrene (see Figures S1 and S2). It is natural to expect that with increasing fraction of DMF in the solvent mixture, more PS chains are dissolved by DMF. However, pure DMF can dissolve and destroy the dimple PS particles (Figure 4). 

**Figure 5:**
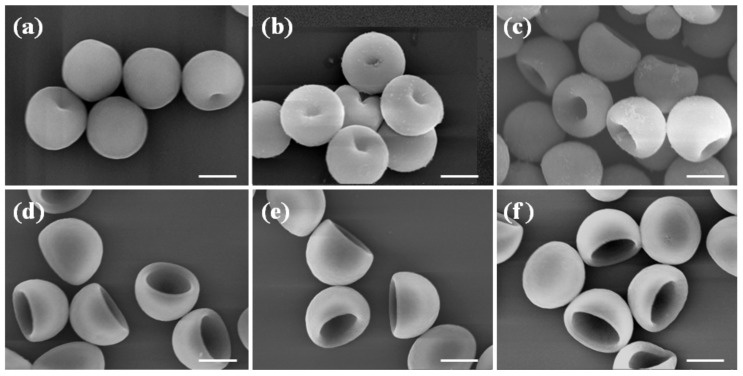
SEM images of PS particles with controlled degree of concaveness. The particles are produced by suspending them in DMF and water mixtures with different volume ratios, *R* = *V*_DMF_/*V*_water_. Form (**a**–**f**), *R* is 0:10, 2:8, 4:6, 6:4, 8:2, 9:1 respectively. Scale bar: 2 μm.

To avoid complete dissolution, instead of using pure DMF we mixed it with water, a poor solvent for the polystyrene. Then by changing the fraction of the good solvent, DMF in the solvent mixture, we can change the amount of non-crosslinked PS chains dissolved and the level of buckling, and hence achieve precise control of the concaveness. The SEM images in Figure 5 demonstrate the successful implementation of the method. As the fraction of DMF increases, the degree of concaveness increases. Meanwhile, the size of the particle and the concaveness remain uniform in each sample.

**Figure 6:**
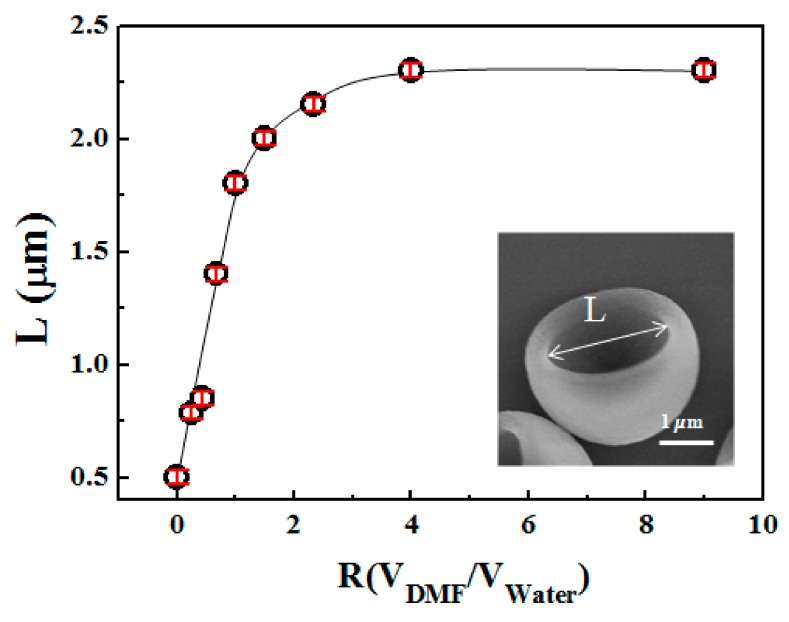
The change of concaveness as a function of DMF volume ratio, *R*, in the DMF/Water mixture. The inset is the SEM image of the concave particles. The maximum length of the open end, *L*, is used to characterize the degree of concaveness. The error bar is the standard deviation of the length. The line is drawn to guide the eye.

To quantify the effect of the DMF/Water mixing ratios on the degree of concaveness, we have used the maximum size of the open end of the concave as an indication of the concaveness. This maximum size is statistically calculated based on the size information obtained from the SEM images. Figure 6 shows the size of concave as a function of the DMF volume ratio (*R* = *V*_DMF_/*V*_water_) in the solvent mixture. The initial size of the concave is 500 ± 20 nm. The overall trend of the size shows that the degree of concaveness increases smoothly and then reached a plateau with increasing *R*. Specifically, when *R* is increased from 0 to 4, the length of the concave reaches 2.3 ± 0.1 μm. Then the size of the concave remains unchanged when further increasing *R* from 4 to 9, (Figure 6). The results can be understood by considering the dissolution power of the solvent mixture. With more DMF in the solvent mixture, more PS chains are dissolved, leading to greater buckling and concaveness. Once all the non-crosslinked PS chains inside the particles are dissolved, no further buckling is possible, so the degree of concaveness stops changing.

### 3.3. Self-Assembly of the Concave Particles

These concave particles, with uniform size and shape, enable novel self-assembly under appropriate conditions. For example, using the concave PS particle as the “lock” and PS sphere or the concave PS particle as the “key,” the self-assembly of lock and key can be induced by depletion interaction between the particles after the addition of an aqueous depletent, PEO. Such depletion interaction is typically attractive and is entropy-driven in nature [30,31]. 

**Figure 7:**
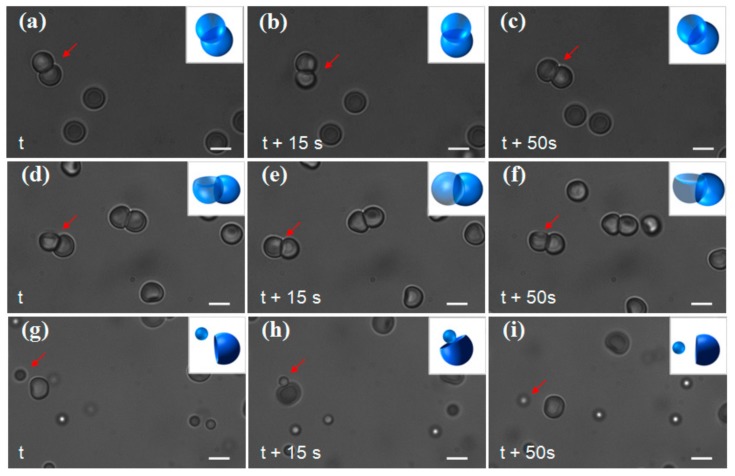
Bright field micrographs of depletion induced assembly. (**a**–**i**) Time-lapse images of the assembly for (**a**–**c**) concave particles with 3 μm PS spheres; (**d**–**f**) concave particles themselves; (**g**–**i**) Time-lapse images of the unsuccessful assembly for concave particles with 1.5 μm PS spheres. The arrows highlight the assemblies or the particles, and the insets illustrate their corresponding configurations. Note the particles and assemblies undergo free motion in all the images, and the change in brightness of particle is the direct result of such motion. Scale bar: 2 μm.

Figure 7 shows time lapse micrographs of the self-assemblies between the lock and the key. The micrographs illustrate that the lock (concave particles with diameter of 3 μm) can readily assemble with the key (PS spheres with diameter of 3 μm, Figure 7a–c), or concave particles with diameter of 3 μm, Figure 7d–f). The typical assembly scenario is that the sphere or the convex side of the key fills into the concave side of the lock. Those self-assembled structure freely move around without falling apart, due to the strong, attractive depletion interaction. However, the lock fails to assemble with smaller PS microsphere (for example, the diameter is 1.5 μm). As show in Figure 7g–i, due to the Brownian motion, one small PS microsphere comes close to the lock (the large concave particle in Figure 7h), but moves away from the lock (Figure 7h), signifying that the depletion is not strong enough to induce the self-assembly.

**Figure 8:**
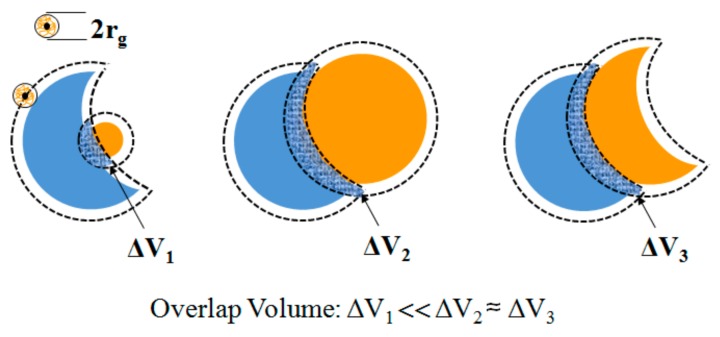
Schematic of the lock–key depletion interaction. Here the lock is the concave particle, and the key are, (**left**) small sphere; (**middle**) large sphere with similar size to the lock and (**right**) the same concave particle as the lock. The depletion interaction depends on size and shape matches between the lock and key, and is proportional to the overlap volume (cross-hatched).

The results demonstrate that the self-assembly relies on the match of size and shape between the concave particle and the other component. In fact, the concept of “lock and key colloids” is proposed based on the size and shape match between colloidal particles [16]. Such self-assembly is typically induced by depletion interaction, which is schematized in Figure 8. The dotted line indicates that each particle surface has a depletion layer with thickness ~r_g_, the radius of gyration of the polymer. When the distance between two particles is less than 2 r_g_, there will be an overlap region of volume ΔV. This overlap will increase the total volume of the polymer in the system and increase the entropy of the polymer. This leads to the decrease of the free energy of the system. The reduction of free energy is ΔF ≈ k_B_Tn_p_ΔV (k_B_ is the Boltzmann constant, T is the absolute temperature, and n_p_ is the polymer number density). When the size and shape of the particles match, the overlap volume is the largest and therefore, and the energy of system reduces the most, inducing the self-assembly. This polymer-induced depletion interaction can also be estimated semi-quantitatively based on the molecular weight and the concentration of polymer in the solution. For a polymer with a molar mass of 600,000 g/mol in its good solvent, the radius of gyration is approximately 50 nm. Together with a PEO concentration of 0.5 g/L, it is possible to estimate the depletion interaction between two spherical particles of 1μm to be approximately 4 k_B_T (see Reference [16] and its Supporting Information for more details). However, in an aqueous system, such as the situation in our experiments, the polymer-induced depletion interaction is of great complexity. The total interaction potential now is a complex function of both electrostatic repulsion and depletion interaction. Nevertheless, our results and previous work [16] have repeatedly shown that such depletion induced self-assembly of concave particles is robust and reproducible, provided similar experimental conditions are met.

## 4. Conclusions

This work demonstrates a simple method to synthesize polystyrene particles with well-controlled degree of concaveness. Uniform PS particles with one single dimple are prepared with seeded emulsion polymerization. Then without utilizing complex chemistry, the degree of concaveness of the dimple PS particle is precisely tuned by a systematic change of the mixing ratio of the good and poor solvents in the solvent mixture. Owing to the well-controlled shape and uniform size, these concave particles are found to self-assemble driven by depletion interaction, which is a significant step towards the fabrication of novel polymeric materials.

## Supplementary Materials

The following are available online at http://www.mdpi.com/2073-4360/10/4/458/s1, Figure S1: FTIR spectrum of the fluffy solid material dissolved by DMF from the dimple particles. Table S1: Assignment of the peaks in the FTIR spectrum. Figure S2: 1H-NMR spectrum of the fluffy solid material dissolved by DMF from the dimple PS particles. Figure S3: SEM images of the concave PS particles. Figure S4: Bright field micrographs of depletion induced self-assembly among concave PS particles. Figure S5: The open length (L) of the concave as a function of the dissolution time. Figure S6: SEM images of PS concave particles. Figure S7: Bright-field microscope images of PS dimple particles dispersed in THF and water mixtures at different volume ratios. Video 1: Assembly of the concave particles with 3 μm PS spheres. Video 2: Assembly of the concave particles with concave particles. Video 3: No assembly for the concave particles with 1.5 μm PS spheres.
