# Supplementary material for: Synthesis of Polystyrene Particles with Precisely Controlled Degree of Concaveness
Source: Polymers (Basel). 2018 Apr 21;10(4):458. doi: 10.3390/polym10040458 (PMC6415454; doi:10.3390/polym10040458)
Supplement: Supplementary file 1 [file polymers-10-00458-s001.zip › polymers-284594-SupplementaryMaterials/Supplementary_Polymers-284594.pdf]

Supplementary Materials

Synthesis of Polystyrene Particles with Precisely Controlled Degree of Concaveness

Wenhua Jing[1], Sinan Du [1], Zexin Zhang[1, 2,] *

[1] Centre for Soft Condensed Matter Physics and Interdisciplinary Research, Soochow University, Suzhou 215006, P. R. China; 18896782986@163.com (W. H. J.); polymerdsn@163.com (S. N. D.)

[2] State and Local Joint Engineering Laboratory for Novel Functional Polymeric Materials, College of Chemistry, Chemical Engineering and Materials Science, Soochow University, Suzhou 215123, P. R. China

* Correspondence: zhangzx@suda.edu.cn; Tel.: +86-512-6915-5295

1. Infrared and NMR Spectra of the Solid Materials Dissolved by DMF from the Dimple Polystyrene Particles

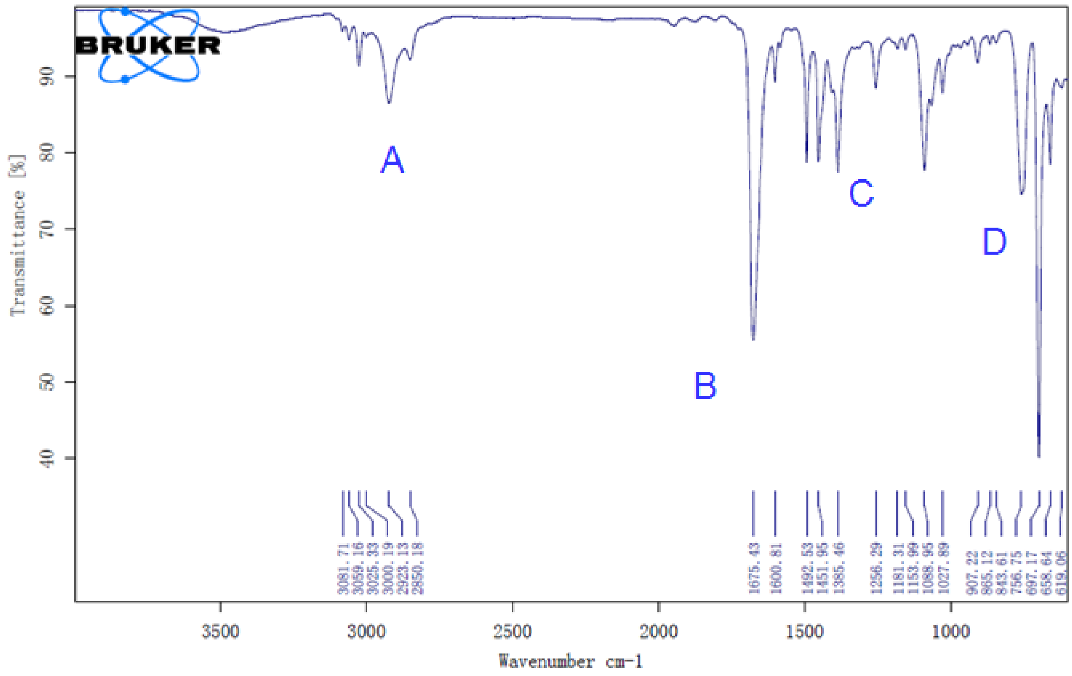

Figure S1. FTIR spectrum of the fluffy solid material dissolved by DMF from the dimple particles. The vibrational bands were labeled with letters A – D, and listed in Table S1 below. The spectrum agrees with previously published results of polystyrene[1].

Region	Wavelength (cm^{-1})	Groups
A	3025	CH
	2923	CH
B	1945	C=C
	1740	C=C
	1600	CH
C	1492	CH benzene ring
	1451	C=C benzene ring
	1385	CH benzene ring
	1088	CH
D	1027	C=C benzene ring
	907	C=C-H
	756	CH benzene ring
	697	CH

Table S1. Assignment of the transmittance peaks in the FTIR spectrum in Figure S1, and they agree with previously published results of polystyrene[1].

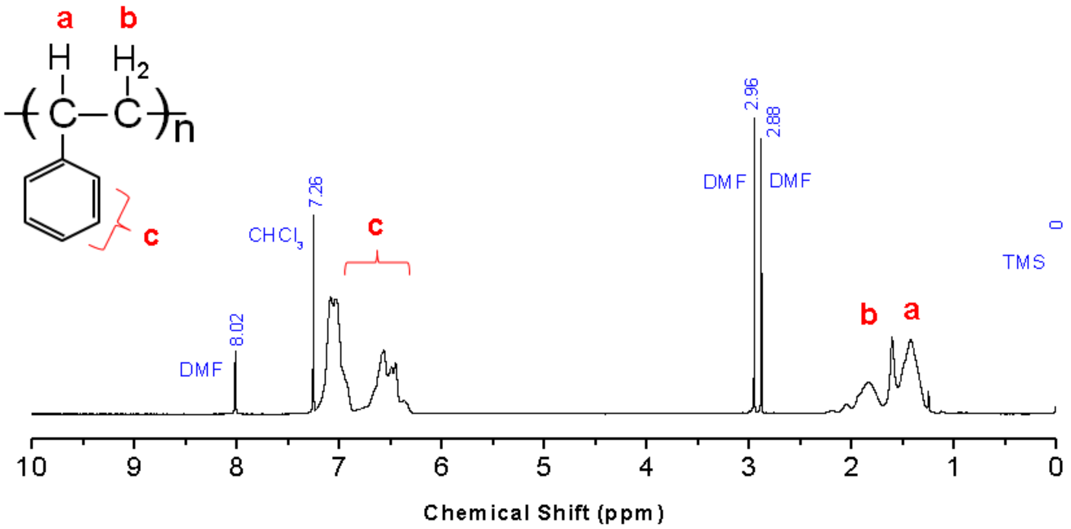

Figure S2. ^{1}H-NMR spectrum of the fluffy solid material dissolved by DMF from the dimple PS particles. The solvent for NMR measurement is $CDCl_3$. The expected resonances from polystyrene are labeled with letters a - c, which are also shown in the structural formula. Signals from the solvent residuals are labeled with the values of their chemical shifts. Note that trace amount of water may also exist in the fluffy solid material and the solvents used at different stages of the particle and sample preparations. The OH proton of water typically contributes a signal at 1.56 ppm when using $CDCl_3$ as the NMR solvent[2].

2. Discussion on Depletion Interaction in Aqueous Systems

The polymer induce depletion interaction can be estimated semi-quantitatively based on the molecular weight and the concentration of polymer in the solution[3, 4]. For PEO polymer with a molar mass of 600.000 g/mol in its good solvent, the radius of gyration is approximately 50 nm. Together with a PEO concentration of 0.5 g/L, it is possible to estimate the depletion interaction between two spherical particles with a diameter of 1 μm to be about 4 k_BT[5]. This estimation is especially accurate for non-aqueous system where electrostatic interaction is typically negligible. However, in an aqueous system, such as the situation in our experiments, the polymer induced depletion interaction is of great complexity[3, 4]. The total interaction potential now is a complex function of both electrostatic repulsion and depletion interaction. For example, PS particles in our work are stabilized with sulfate surface groups, which dissociate in water and cause the particles to be negatively charged. The acid-cleaned glass surface of the sample cell is also highly negatively charged. And thus the interaction between PS particles and glass surface is dominated by electrostatic repulsion and is long-ranged in nature. Actually it has been reported that even when the distance between a PS particle and a glass surface is 300 nm, the electrostatic repulsion can still be effective (~ 4 k_BT)[5]. By comparison, the polymer-induced depletion interaction is generally short-ranged and the range of interaction is determined by the size of the polymer. In our work the radius of gyration of PEO is approximately 50 nm. As a result, the long-range repulsion (~ 300 nm) can prevent the particle coming close to the glass surface, and the depletion can hardly become effective. That's probably why we never observed any particle stuck on glass slide due to the depletion interaction. A more quantitative estimation of the deletion interaction is desirable but challenging due to the following: (1) In an aqueous system, the total interaction potential (depletion + electrostatic) is complex, as discussed above; (2) The depletion interaction itself in an aqueous system is a complex function of the polymer concentration due to different concentration regimes of a polymer solution; (3) The concave shape makes the situation more complicate than those of spheres and/or flat surfaces; (4) The surface roughness of the concave particle make the estimation of deletion interaction even more challenging. For example, after close inspection of the concaved particles, we found that the surface of the concaved particle are rough, with a root mean squared roughness on the order of tens of nanometers (Figure S3). This roughness makes the accurate determination of the depletion difficult because previous theoretical and experimental studies have shown that roughness greatly affects the depletion interaction [6, 7].

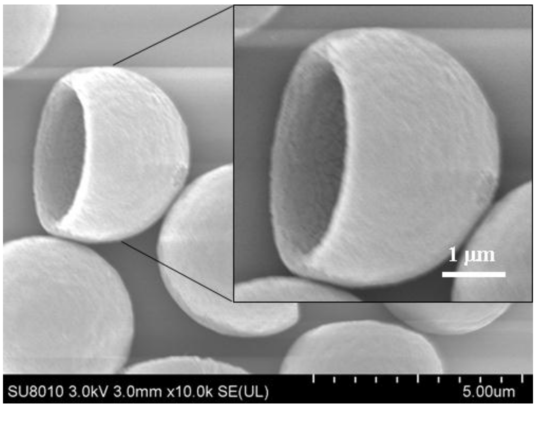

Figure S3. SEM images of the concave PS particles. Note that both the interior and exterior surfaces are rough.

3. Additional Images of the Depletion Induced Assembly

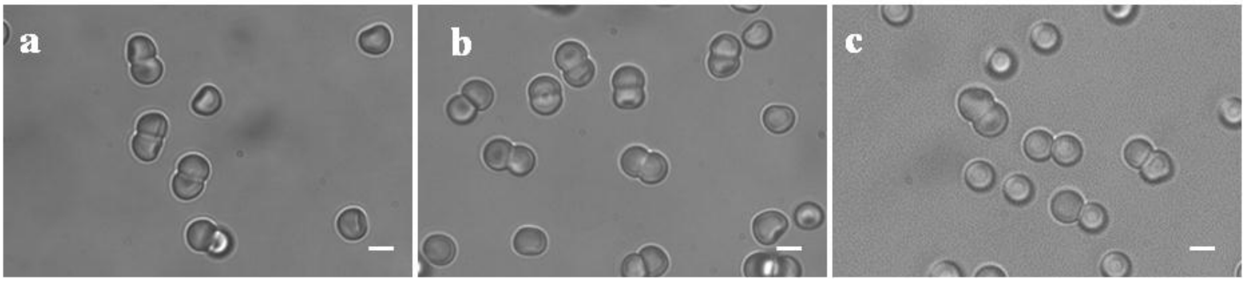

Figure S4. Bright field micrographs of depletion induced self-assembly among concave PS particles. Scale bar: 2 μm

4. Notes on the Dissolution of Polymer

Polymer dissolution has been of great interest for both academia and industry. It has been characterized and understood over the years. Compared with that of a small molecule, the dissolution of polymer, even non-crosslinked one, takes days to weeks. It has been reported that there are several steps during polymer dissolution: solvent penetration, formation of swollen gel layer, formation of liquid layer and chain disentanglement, to name a few[9]. As expected all the steps take long time. In our studies, in order to induce buckling, the polymer chains inside the particle need to be not only dissolved, but also transported by the solvent reflux to the surface of particle and removed later by washing. In addition, the surface layer of the particle is crosslinked due to the slow addition of the cross-linker. This crosslinked layer makes the penetration of solvent and the late transportation of chains through the layer even more difficult. By trial and error we have found that 21 days of dissolution time always yields consistent and reproducible results, regardless of the mixing ratios of the water and DMF.

4.1 Effect of Dissolution Time on the Formation of Concave

We chose DMF and water mixture at high volume ratio, V_{DMF}:V_{water} = 4, to investigate the effect of dissolution time on the formation of concave. We found that the size of the concave increases when increasing the dissolution time, and the size stops increasing after 12 days, indicating the dissolution reaches equilibrium. (Figure S5).

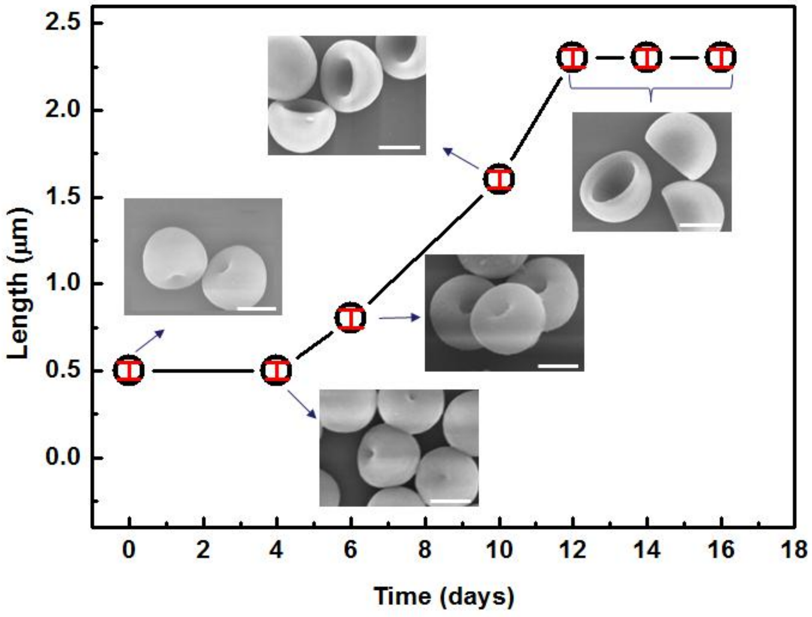

Figure S5. The open length, *L*, of the concave versus the dissolution time. The insets show the corresponding SEM images. Scale bar: 2 μm.

4.2 Other Solvents for the Dissolution of Polystyrene

We have performed preliminary studies on other water-miscible organic solvents. For example, tetrahydrofuran (THF) and acetone were tested to form concave particle. The DMF was changed to THF or acetone, and the same dissolution and wash procedures were carried out. The dimple PS particles were found to form concave when dispersed in those solvent mixtures (Figure S6), but only at very low percentage of organic solvents. At high mixing ratio of the organic solvents, the dimple particles are found to either coalesced or aggregated, possibly due to the plasticize (soften) effect of the organic solvents, as reported previously[10] (Figure S7). These results can be rationalized by considering the solubility parameter of PS and the organic solvents. The solubility parameters of THF and acetone are closer to PS than that of DMF, so THF and acetone can dissolve the PS much more effectively, leading to the coalescence or aggregation of the PS particles.

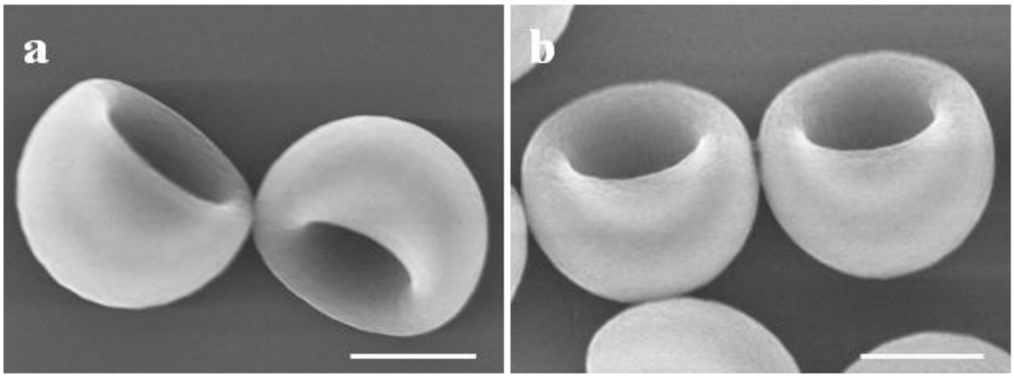

Figure S6. SEM images of PS concave particles. The particles are dispersed in the following solvent mixtures for 4 days and then washed by deionized water for 3 times: (a) THF and water mixture at the volume ratio, $V_{THF}:V_{water}$ = 0.1, (b) acetone and water mixture at the volume ratio, $V_{acetone}:V_{water}$ = 0.25, Scale bar : 2μm.

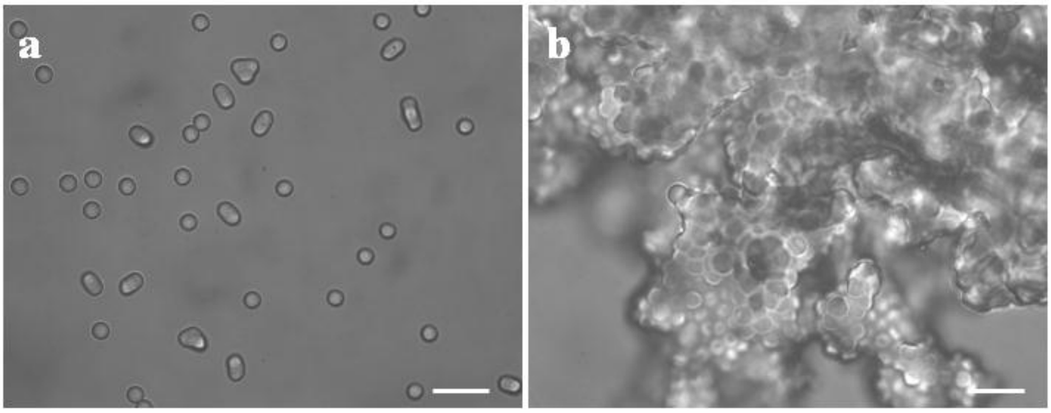

Figure S7. Bright-field microscope images of PS dimple particles dispersed in THF and water mixtures at different volume ratios for 12 hours, (a) $V_{THF}:V_{water}$ = 1, (b) $V_{THF}:V_{water}$ = 4. The particles are either coalesced or aggregated. Scale bar: 10 μm.

5. Supporting Videos of the Depletion Induced Assembly

Video 1. Assembly of the concave particles with 3 μm PS spheres.
Video 2. Assembly of the concave particles with concave particles.
Video 3. No assembly for the concave particles with 1.5 μm PS spheres

Note all the video are 90 seconds, and are played at 5 frames per second.

References

[1] Liang, C. Y.; and Krimm, S. Infrared Spectra of High Polymers. VI. Polystyrene. *Journal of Polymer Science*, **1958**, *27*, 241–254

[2] Fulmer, G. R.; Miller, A. J. M.; Sherden, N. H. ;Gottlieb, H. J.; Nudelman, A.; Stoltz, B. M.; Bercaw, J. E.; Goldberg, K. I. NMR Chemical Shifts of Trace Impurities: Common Laboratory Solvents, Organics, and Gases in Deuterated Solvents Relevant to the Organometallic Chemist. Organometallics **2010**, *29*, 2176–2179

[3] Asakura, S., &. Oosawa, F. On Interaction Between Two Bodies Immersed in a Solution of Macromolecules. *J. Chem. Phys.* **1954**, *22,* 1255–1256.

[4] Tuinier, R.; Vliegenthart, G. A.; and Lekkerkerker, H. N. W. Depletion Interaction Between Spheres Immersed in a Solution of Ideal Polymer Chains. *J. Chem. Phys.* **2000**,*113*,10768.

[5]. Sacanna, S.; Irvine, W. T. M.; Chaikin, P. M.; & Pine, D. J. Lock and Key Colloids, *Nature*, **2010**, *464*, 575-579.

[6] Bechinger, C.; Rudhardt, D.; and Leiderer, P. Understanding Depletion Forces beyond Entropy, *Phys. Rev. Letts.* **1999**, *83,* 3960-3964.

[7] Zhao, K.; and Mason, T. G. Directing Colloidal Self-Assembly through Roughness-Controlled Depletion Attractions, *Phys. Rev. Letts.* **2007**, *99,* 268301.

[8] Kraft, D. J.; Ni, R.; Smallenburg, F.; Hermes, M.; Yoon, K.; Weitz, D. A.; van Blaaderen, A.; Groenewold, J.; Dijkstra, M.; Kegel, W. K., Surface roughness directed self-assembly of patchy particles into colloidal micelles. *Proc. Natl. Acad. Sci. U.S.A.* **2012**, *109,* 10787-10792.

[9] Miller-Chou, B. A.; Koenig, J. L. A Review of Polymer Dissolution. *Prog. Polym. Sci.* **2003**, *28*, 1223–1270.

[10] Liu, B.; and Wang, D. Y. High-Throughput Transformation of Colloidal Polymer Spheres to Discs Simply via Magnetic Stirring of Their Dispersions. *Langmuir* **2012**, *28*, 6436−6440.
